# Advancing humanitarian assistance amid adversity in 2025

**DOI:** 10.2471/BLT.25.293672

**Published:** 2025-08-01

**Authors:** Abdullah A Al Rabeeah, Shahul Ebrahim, Barrak Alahmad, Abdullah Almoallem, Ziad A Memish

**Affiliations:** a King Salman Humanitarian Aid and Relief Center, P.O. Box 12371, Riyadh 7046, Saudi Arabia.; b School of Medicine, University of Science, Technique and Technologies of Bamako, Mali.; c Harvard T.H. Chan School of Public Health, Harvard University, Cambridge, United States of America.

In February 2024, the Lobito Corridor, a 1300 km railway linking Angola, the Democratic Republic of the Congo and Zambia to global markets, neared completion.^1^ However, in January 2025, conflict escalated in Goma, the Democratic Republic of the Congo, a key geostrategic hub of the railway, stalling development in the country.^2^ This example illustrates how local flare-ups in conflict environments can reignite long-standing crises and cause regional instability. 

According to the Uppsala Conflict Data Program, the global number of State-based armed conflicts has steadily increased since 2010.^3^ This global conflict burden has contributed to five major stressors that challenge the humanitarian architecture and require enhanced coordination among donors, nongovernmental organizations (NGOs) and governments.

First, the global humanitarian system is facing its most important financing crisis since the establishment of the United Nations (UN). Humanitarian aid budgets declined from approximately 42 billion United States dollars (US$) in 2022 to US$ 32 billion in 2024.^4^ Major donors have announced further reductions, citing domestic fiscal constraints and shifting geopolitical priorities.^5^

In 2025, the Organisation for Economic Cooperation and Development (OECD) projected a 14% decline in official development assistance to fragile and conflict-affected countries.^4^ This underfunding has created programmatic gaps in child nutrition, cholera prevention and maternal health across conflict-affected regions. The multilateral system is further weakened by a lack of donor coordination, duplicated NGO efforts and limited engagement with local civil society partners.

Second, the number of forcibly displaced people reached 122 million globally in 2024.^6^ Three quarters of these refugees were hosted in low-income countries, placing immense strain on already fragile national systems. 

Third, a strong correlation exists between weak health governance, conflict and infectious disease outbreaks. Multiple novel and re-emerging pathogens appeared in 2024 and early 2025: mpox (Clade I and II), Nipah virus, Chandipura virus, Oropouche virus, and ongoing threats from Middle East Respiratory Syndrome (MERS), coronavirus (COVID-19) and influenza virus variants.^7^^,^^8^ Cholera affected 33 countries in 2024, with the highest burden in war-torn nations. However, no coordinated cross-agency early warning system is yet in place across conflict zones.

Fourth, 733 million people were undernourished in 2023, up by 152 million from 2019.^9^ This increase is driven by climate shocks, conflict and trade disruptions. World Food Programme reports show that funding cuts led to ration reductions in 19 countries in late 2024, raising the risk of famine,^10^ yet food systems are still addressed in isolation. 

Finally, the burden of climate change is increasingly evident. In 2024, heatwaves in India, typhoons in south-east Asia, wildfires in Algeria and Greece, and floods in Brazil and Kenya displaced over 15 million people. Extreme weather is now a key driver of forced migration and crop failures, yet early warning systems and anticipatory finance mechanisms remain underutilized.

Despite these challenges, the global humanitarian architecture lacks a unified mechanism to systematically track funding and operational gaps, particularly those arising in conflict-affected settings. A strategy that can identify needs in real time, harmonize donor contributions and embed national and local actors into every stage of operational planning is needed. This strategy should prioritize conflict-sensitive needs mapping, leveraging platforms such as the humanitarian data systems of the UN Office for the Coordination of Humanitarian Affairs (OCHA), while ensuring local validation. A global funding gap task force, operating under the Inter-Agency Standing Committee, could serve to coordinate donor commitments, monitor shortfalls and guide equitable resource allocation. Simultaneously, embedded government coordination hubs in affected and host countries are essential to ensure national ownership and alignment with domestic systems. The deployment of accessible real-time programmatic dashboards would enhance transparency, facilitate joint decision-making and reduce duplication. Finally, institutionalized learning platforms, such as the Riyadh International Humanitarian Forum (RIHF), should be used as mechanisms to evaluate progress, promote cross-sector alignment and ensure that humanitarian action remains coherent, adaptive and accountable. The 4th RIHF,^11^ convened by KSrelief in collaboration with the UN in February 2025, addressed these five stressors with the objective of advancing humanitarian assistance amid adversity. The forum brought together 12 UN agencies and 150 organizations, including universities and NGOs, and featured 132 global experts across 21 panels. The 5th RIHF, scheduled for 2027, will continue to focus on the five major stressors outlined here as part of a broader agenda to institutionalize forward-looking humanitarian responses.

To address today’s realities of conflict, climate change and disease spread, the coordination between donors, NGOs and governments must be recalibrated, not just to fund response, but to shape a unified global humanitarian strategy rooted in equity, efficiency and foresight.

## References

[1] The Lobito Corridor: building Africa’s most important railway. Washington, DC: US Chamber of Commerce; 2025. Available from: https://www.uschamber.com/international/the-lobito-corridor-building-africas-most-important-railway [cited 2025 Mar 4].

[2] DR Congo crisis: Security Council hears call for ‘urgent and coordinated international action’ over Goma. New York: United Nations; 2025. Available from: https://news.un.org/en/story/2025/01/1159511 [cited 2025 Mar 4].

[3] Number of conflicts 1975-2023. Uppsala: Uppsala Conflict Data Program; 2023. Available from: https://ucdp.uu.se/ [cited 2025 Mar 4].

[4] OECD policy brief - cuts in official development assistance OECD projections for 2025 and the near term. Paris: Organisation for Economic Cooperation and Development; 2025. Available from: https://data.unhcr.org/en/documents/details/117285 [cited 2025 Jul 8].

[5] Gulrajani N, Pudussery J. With the knives out on development spending, have we reached ‘peak aid’? The Guardian. 2025 Jan 23. Available from: https://www.theguardian.com/global-development/2025/jan/23/global-development-economics-donor-spending-refugee-oecd-world-bank-peak-aid [cited 2025 Jul 7].

[6] Figures at a glance. Geneva: United Nations High Commissioner for Refugees; 2025. Available from: https://www.unhcr.org/uk/about-unhcr/overview/figures-glance [cited 2025 Jul 7].

[7] WHO SEAR 15th Epidemiological Bulletin 2024. Geneva: World Health Organization; 2024. Available from: https://www.who.int/publications/i/item/9789290211686 [cited 2025 Jul 7].

[8] Emerging infectious diseases: global trends in zoonotic and respiratory viral threats – Annual Report 2024. Atlanta: Centers for Disease Control and Prevention; 2025. Available from: https://www.cdc.gov/global-health/annual-report-2024/index.html [cited 2025 Jul 7].

[9] The state of food security and nutrition in the world 2024. Rome: Food and Agriculture Organization of the United Nations; 2024. Available from: https://www.fao.org/publications/home/fao-flagship-publications/the-state-of-food-security-and-nutrition-in-the-world/en [cited 2025 Feb 1].

[10] The state of food security and nutrition in the world. Financing to end hunger, food insecurity and malnutrition in all its forms. Rome: Food and Agriculture Organization of the United Nations; 2024. Available from: https://openknowledge.fao.org/server/api/core/bitstreams/d5be2ffc-f191-411c-9fee-bb737411576d/content [cited 2025 Jul 8].

[11] Fourth Riyadh international Humanitarian Forum. Riyadh: KSrelief. 2025. Available from: https://rihf.ksrelief.org/ [cited 2025 Mar 4].

